# Quantifying Transmission Heterogeneity Using Both Pathogen Phylogenies and Incidence Time Series

**DOI:** 10.1093/molbev/msx195

**Published:** 2017-07-11

**Authors:** Lucy M. Li, Nicholas C. Grassly, Christophe Fraser

**Affiliations:** 1 Department of Infectious Disease Epidemiology, School of Public Health, Imperial College London, London, United Kingdom; 2 Center for Communicable Disease Dynamics, Harvard T.H. Chan School of Public Health, Boston, MA; 3 Nuffield Department of Medicine, Oxford Big Data Institute, University of Oxford, Oxford, United Kingdom

**Keywords:** phylodynamics, infectious disease, parameter inference, polio

## Abstract

Heterogeneity in individual-level transmissibility can be quantified by the dispersion parameter k of the offspring distribution. Quantifying heterogeneity is important as it affects other parameter estimates, it modulates the degree of unpredictability of an epidemic, and it needs to be accounted for in models of infection control. Aggregated data such as incidence time series are often not sufficiently informative to estimate k. Incorporating phylogenetic analysis can help to estimate k concurrently with other epidemiological parameters. We have developed an inference framework that uses particle Markov Chain Monte Carlo to estimate k and other epidemiological parameters using both incidence time series and the pathogen phylogeny. Using the framework to fit a modified compartmental transmission model that includes the parameter k to simulated data, we found that more accurate and less biased estimates of the reproductive number were obtained by combining epidemiological and phylogenetic analyses. However, k was most accurately estimated using pathogen phylogeny alone. Accurately estimating k was necessary for unbiased estimates of the reproductive number, but it did not affect the accuracy of reporting probability and epidemic start date estimates. We further demonstrated that inference was possible in the presence of phylogenetic uncertainty by sampling from the posterior distribution of phylogenies. Finally, we used the inference framework to estimate transmission parameters from epidemiological and genetic data collected during a poliovirus outbreak. Despite the large degree of phylogenetic uncertainty, we demonstrated that incorporating phylogenetic data in parameter inference improved the accuracy and precision of estimates.

## Introduction

The intensity of epidemics is often summarized by the reproductive number R, the average number of secondary infections caused by a typical infectious individual over the course of their infectious period. This statistic is useful for determining whether an epidemic can take off and if so the final size of the epidemic. However, large variation between individuals is frequently observed in outbreaks of directly transmitted acute infections leading to superspreading events such that a few individuals cause most of the infections (Lloyd-Smith etal. 2005). The offspring distribution captures the distribution of secondary infections per infectious individual, and can be parameterized by a negative binomial with mean R and dispersion k. The presence of superspreading as indicated by small values of k can affect the effectiveness of control strategies (Garske and Rhodes 2008).

Inferring the value of k from data is not straightforward, even in the presence of contact tracing data as many infections may be asymptomatic or not reported. The offspring distribution fit to incomplete transmission chain data has to be corrected for biased and under-reporting (International Ebola Response Team 2016). Obtaining precise estimates of k from just incidence time series is usually not possible because k only affects the noisiness of the incidence time series at low numbers.

Besides epidemiological data, pathogen population genetics are playing an increasingly important role in inferring epidemiological parameters (Volz etal. 2009; Koelle and Rasmussen 2012; Volz 2012; Stadler etal. 2013; Kühnert etal. 2014). For coalescent-based approaches, the offspring distribution is integral to the inference process as it affects the relationship between the underlying epidemic and the observed distribution of coalescent (branching) events in the pathogen phylogeny. When the offspring distribution is overdispersed, shorter intervals between coalescent times in the pathogen phylogeny are observed. This is expected as coalescent events correspond to transmission events during the epidemic and superspreaders can cause the aggregation of many transmission events within a short period of time.

Given that epidemiological parameters could be estimated either from epidemiological data or from phylogenetic data, combining the analysis of both types of data should provide more accurate and precise estimates. Rasmussen etal. (2011) found that estimating parameters jointly from both incidence time series and pathogen phylogeny reduced uncertainties in estimates of parameters and the prevalence over time. However, this work did not allow for uncertainty in the pathogen phylogeny and was limited to simple SIR models. While Rasmussen etal. (2014) showed that parameter estimates did not significantly change for phylogenies of sequences collected over many years, the uncertainty in pathogen phylogeny during outbreaks is generally greater and needs to be accounted for to ensure accurate estimation of transmission parameters.

Here we develop a statistical inference framework to fit a stochastic compartmental model with an explicit offspring distribution to enable the estimation of k and other epidemiological parameters from outbreak data when there is uncertainty in the pathogen phylogeny. A major challenge is that small numbers of infections at the start of epidemics combined with highly heterogeneous transmission lead to substantial stochasticity in the initial dynamics of epidemics. We used the Particle Monte Carlo Markov Chain (PMCMC) method to infer parameters while integrating over stochastic outcomes (Andrieu etal. 2010; Rasmussen etal. 2011).

We simulated data to assess the coverage, precision, and bias of estimates of k and other epidemiological parameters using our method. We also applied our inference framework to genetic sequence and epidemiological data collected during a poliovirus outbreak (Yakovenko etal. 2014). Although polio sequences have provided interesting insight into pathogen diversity and geographic transmission routes, phylodynamic methods such as those presented here have not previously been used to infer epidemiological parameters.

## New Approaches

The inference framework that we developed here uses both incidence time series and pathogen phylogenies to infer the value of k and other epidemiological parameters. It is based on integrating results from coalescent theory and epidemiological modeling into a combined inference framework and using particle filtering to estimate the marginal likelihood of the data under the model in an MCMC approach. The main novelty of our method is the ability to estimate k by fitting stochastic compartmental models to both epidemiological and phylogenetic data. To do this we needed a transmission model with an explicit offspring distribution, a formulation of the coalescent parameterized in terms of the offspring distribution parameters, and a method to integrate over the phylogenetic uncertainty that often arises in rapid outbreaks.

### Compartmental Model with Explicit Offspring Distribution

The offspring distribution describes the distribution of secondary infections $Z_{i}$ caused by each infectious individual i. The offspring distribution for unstructured compartmental models such as the SIR is geometric due to the Poisson transmission process and exponentially distributed duration of infection. However, the geometric is not able to capture the variation in infectiousness for several directly transmitted diseases for which the negative binomial provides a better fit (Lloyd-Smith etal. 2005). We use a negative binomial distribution with mean $R_{t}$ and variance $R_{t}(1+\frac{1}{k})$ to characterize the offspring distribution of individuals infected at time step $t=\{1,\ldots ,n_{T}\}$, where the size of the simulation time step is Δt. The dispersion parameter k determines the level of overdispersion in the offspring distribution. A small k means that a few superspreading individuals cause most of the infections.

Without specifying an offspring distribution, the implicit assumption of the stochastic SIR model is that the offspring distribution is geometric. Throughout this paper, we parameterize the negative binomial with the mean and dispersion as they are the most relevant statistics for epidemiological studies. For a given individual i infected at time step $H_{i}$, the number of secondary infections they cause is distributed according to $Z_{i}∼NBin(R_{t=H_{i}},k)$. The reproductive number $R_{t}$ is the average number of secondary infections caused by an individual infected at time step t: $R_{t}=E(Z_{i}|H_{i}=t)$.

We simulated from a modified susceptible-infected-removed (SIR) model using the binomial distribution based tau-leap method (Chatterjee etal. 2005). As we focused on the analysis of acute infectious diseases with short generation times, we approximated the transmission process by assuming all secondary infections occur at the end of the infectious period. This approximation was used so that we could simulate according an arbitrary offspring distribution without having to keep track of infection times of infected individuals. In practice, this meant drawing a random number from $\nu_{i}∼NBin(R_{t=H_{i}}Y_{t},kY_{t})$, where $Y_{t}$ was the number of infectious individuals recovering at time step *t*.

### Coalescent Likelihood

The original coalescent provided the statistical distribution of coalescent times for a given effective population size Ne (Kingman 1982). In the context of epidemiology, Ne is related to the number of infectious individuals N via the variance of the offspring distribution $\sigma^{2}$ at endemic equilibrium $Ne=\frac{N}{\sigma^{2}}$ (Koelle and Rasmussen 2012). The discrepancy between the total number of infectious individuals and the effective number of individuals contributing to infections increases with the variance of the offspring distribution. For infectious diseases, N corresponds with the number of infectious individuals Y (time index t has been dropped for clarity).

Going backward in time, the probability density function of the time to coalescence of a pair of lineages U is $f(U)=\lambda e^{-\lambda U}$, where rate $\lambda =\frac{1}{Ne\cdot T_{g}}$ and $T_{g}$ is the generation time (duration of infection).

In this work, we use a formulation of the coalescent parameterized by an arbitrary offspring distribution that is time-varying (Fraser and Li 2017). Assuming a negative binomial offspring distribution with mean R and variance $R(1+\frac{R}{k})$, the expected rate of coalescence for a pair of lineages is given by:

(1) $$\begin{array}{c} \lambda =\frac{R(1+\frac{1}{k})}{N\cdot T_{g}} \end{array}$$,

where $T_{g}$ is the mean generation time, that is, the duration of infectiousness in an SIR model. We assume that λ,R,andN change over time in a step-wise fashion at each simulation time step. Equation (1) is used to calculate the likelihood given a phylogeny (see Materials and Methods for more details).

### Phylogenetic Uncertainty

To avoid the computationally intensive process of phylogenetic reconstruction on top of particle filtering, we separated the process of parameter estimation and phylogeny reconstruction. In Rasmussen etal. (2014), the authors used the same approach but with fewer (ten) phylogenies and they did not pool together posterior estimates to get an overall posterior distribution. An alternative approach was taken by Volz and Pond (2014) where they calculated the average likelihood across all phylogenies at each iteration of MCMC. Because we are adopting a PMCMC approach, it was more computationally efficient to run parallel PMCMC for each phylogeny than using the particle filter multiple times at each iteration of the MCMC.

Phylogenetic reconstruction programs such as MrBayes (Ronquist etal. 2012) produce a posterior distribution P(Phy|S) of phylogenies given the sequences S. To estimate the marginal posterior probability of the parameters P(θ|S), we can integrate over the phylogenies: $P\left(\theta |S\right)\propto P(\theta )\int_{\text{Phy}}^{}P(\text{Phy}|\theta )P(\text{Phy}|S)d\text{Phy}$. Taking *M* samples from the posterior distribution P(Phy|S), we can estimate the marginal posterior density P(θ|S) using equation (2).

(2) $$P\left(\theta \text{|}S\right)\propto \frac{P\left(\theta \right)}{M}\sum_{m=1}^{M}P({\text{Phy}}^{\left(m\right)}|\theta ).$$

However, as we reconstruct the phylogeny independently from estimation of epidemiological parameters, equation (2) is only an approximation of the marginal posterior density. The number of phylogenies M needed to accurately estimate epidemiological parameters depends on uncertainties in branching times and the molecular clock rate.

### Implementation

Although programs that implement PMCMC do exist, they are mostly tailored for analyses of incidence time series (Dureau etal. 2013; King etal. 2016). Existing code for concurrently analyzing incidence time series and phylogenetic data is not parallelized and does not allow for phylogenetic uncertainty (Rasmussen etal. 2014). Parallelization is not needed if the number of particles is small (a few hundred), for example for analyzing data generated through largely deterministic processes. The number of particles needed to obtain stable estimates of likelihood scales with the length of the time series (Andrieu etal. 2010) as well as the stochasticity of the model. Without parallelization, the MCMC would not converge in a reasonable amount of time for outbreak data.

We implemented a parallelized version of particle filtering implemented in C ++ (code available at github.com/lucymli/EpiGenMCMC; last accessed June 23, 2017) with an accompanying R package (github.com/lucymli/EpiGenR; last accessed June 23, 2017) to interface with the C ++ program. More details on the implementation are provided in the “Materials and Methods” section.

## Results

An overview of the simulated data analyzed in the following sections is provided in the “Materials and Methods” section.

### Phylogeny Is More Informative than Incidence Time Series for Estimating k

**Fig. 1.**
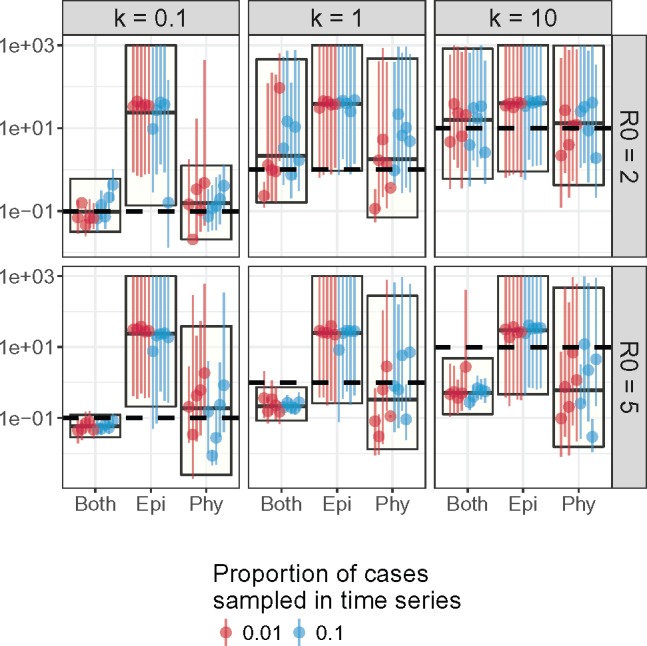
Estimates of *k* from simulated data. The horizontal lines denote the true value of *k* for that set of parameters, that is, the value used to generate the simulated data. The boxes with a horizontal line in the middle indicate the median and 95% HPD interval of parameter estimates pooled from all simulations for that parameter set. The vertical lines with a single dot denote the median and 95% HPD interval of each individual simulation. Blue lines are from simulations in which 10% of individuals were sampled. Red lines are from simulations in which 1% of individuals were sampled.

Based on data simulated from an SIR model, pathogen phylogeny was needed to accurately estimate the dispersion parameter k of the offspring distribution when k was small (fig. 1). This suggested that superspreading events left sufficient signal to allow inference of k in the phylogeny but not the incidence time series. There was insufficient signal in the incidence time series to determine the value of k. Although using both epidemiological and phylogenetic data produced the least biased and most precise estimates of k, the coverage was lower than using each set of data alone as the true value did not fall within the credible intervals in many instances. In fact, using phylogenetic data alone seemed to produce the most accurate estimates that were only slightly less precise and slightly more biased compared with estimates that used both types of data (table 1).

**Table 1.** Precision (measured by the root mean squared deviation), Bias, and Coverage (% of simulations in which the true value is found in the 95% highest posterior density intervals) of Parameter Estimates When Fitting Models to Either Epidemiological, Phylogenetic, or Both Types of Data.

*Data*	$R_{0}$*: RMSD*	$R_{0}$*: Bias*	$R_{0}$*: in HPD*	k*: RMSD*	k*: Bias*	k*: in HPD*
Both	0.1507	−0.0756	100%	15.0737	0.0103	75%
Epi	0.1695	−0.0387	100%	673.9374	93.4255	100%
Phy	0.1863	−0.0855	100%	31.7680	0.5461	100%
*Data*	$T_{0}$*: RMSD*	$T_{0}$*: Bias*	$T_{0}$*: in HPD*	ρ*: RMSD*	ρ*: Bias*	ρ*: in HPD*
Both	11.0638	0.1813	100%	0.1183	−0.0766	75.00%
Epi	11.4962	0.3933	100%	0.1267	−0.1002	83.33%
Phy	11.9352	−1.2768	100%	NA	NA	NA

Note.—These statistics were evaluated across all simulations presented in figure 1. The estimates of epidemic start dates $T_{0}$ were converted to the number of days after an arbitrary date. For bias and precision, we normalized the statistics by the true parameter value.

We also estimated the basic reproductive number $R_{0}$, time of the first infection $T_{0}$, and the probability of sampling an infectious individual in the incidence time series ρ at the same time as estimating k. There were no noticeable differences between estimates of these parameters when only epidemiological, only phylogenetic, or both data sets were used for inference (supplementary fig. S1, Supplementary Material online). We did not estimate ρ when just using the phylogenetic data, as the reporting rate ρ referred to the probability that an infection appeared in the incidence time series.

Estimates of the $R_{0}$ and k were closer to the true value when both genetic and epidemiological data were used in inference, compared with fitting to each set of data individually (table 1). The coverage of estimates for $T_{0}$ and ρ while fitting to both sets of data was similar to fitting to epidemiological or phylogenetic data alone.

### Estimates of $R_{0}$ Were Biased If k Was Fixed at the Incorrect Value

The dispersion parameter k of the offspring distribution is usually not estimated when fitting compartmental models. We investigated the effects of assuming the wrong value of k on parameter estimates, especially on $R_{0}$ estimates. The implicit assumption of an unstructured SIR compartmental transmission model is that the offspring distribution is geometrically distributed, which is equivalent to fixing k=1 in the negative binomial. For a subset of simulated outbreaks (those where we sampled 1% of individuals), we re-estimated parameters with a fixed k=1.

**Fig. 2.**
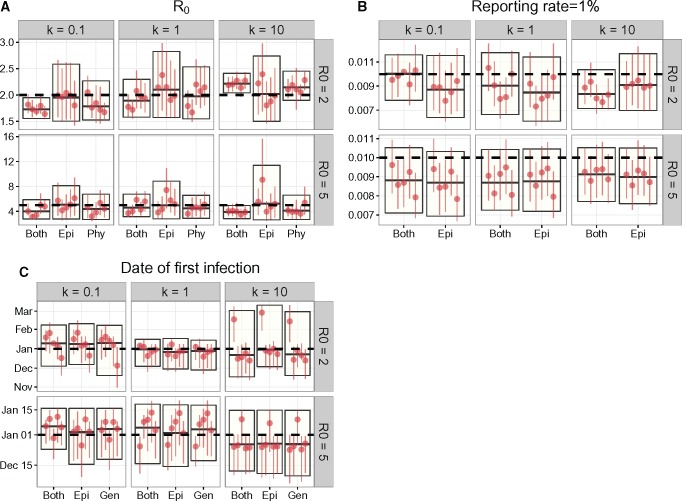
Parameter estimates when reporting rate was 1 in 100 and *k* was fixed to 1. The horizontal dashed lines denote the true parameter value for that set of parameters that is, the parameter value used to simulate the data. The boxes indicate the median and 95% HPD interval of parameter estimates pooled from replicate simulations. The vertical lines with a single dot denote the median and 95% HPD interval of each individual simulation. Simulations where the MCMC chain did not converge were left out of the plot. Estimates of the reporting rate did not include inference from phylogenetic data, as the reporting rate refers to the probability that an infection appears in the incidence time series.

We compared these results (fig. 2) to those obtained when k was also estimated (fig. 1), and found significant differences in $R_{0}$ estimates when the true value of k≠1. This was evidenced by an increase in the Kolmogorov–Smirnov distances (Massey 1951), a measure of distance between two distributions when the true value of k≠1 compared with when the true value of k=1 (table 2). Inference using just incidence time series was less affected by assumptions of k, given the small K–S distances.

**Table 2.** The Kolmogorov–Smirnov (K–S) Distance between the Posterior Distributions of $R_{0}$ Estimated Assuming a Geometric Offspring Distribution (i.e., fixing k=1) and Those Estimated While Estimating k (see results in table 1 and figure 1).

k	*Both*	*Epi*	*Phy*
0.1	0.725 (0.078, 0.956)	0 (0, 0.125)	0.201 (0, 0.887)
1.0	0.325 (0.111, 0.979)	0.151 (0.078, 0.247)	0.422 (0.142, 0.777)
10.0	0.646 (0.135, 0.977)	0.066 (0, 0.101)	0.395 (0.067, 0.954)

Note.—K–S values closer to 1 reflect larger discrepancies between the posterior distributions, whereas those close to 0 suggest no difference in posterior distributions. The numbers in the brackets denote the range (maximum–minimum) of K–S distances from different sets of simulated data, and the number preceding the brackets denotes the median K–S distance.

**Table 3.** Parameters of the SIR Model Fit to Simulated Outbreak Data.

Parameter	Value	Prior
Population size $N_{\text{Total}}$	20,000	—
Initial number of infected $I_{0}$	1	—
Duration of infection $T_{g}=\frac{1}{\gamma }$	—	Uniform (3, 7)
Basic reproductive number$R_{0}$	—	Uniform (1, 20)
Offspring distribution dispersion k	—	$\frac{1}{k}∼$ Uniform $(1\times 10^{-4}$, $1\times 10^{4}$)
Reporting rate ρ	—	Uniform (0.0, 1.0)
Time of first infection $T_{0}$	—	Uniform (01 Jan 16, ⋅)

Note.—The upper bound of the prior distribution of the epidemic start date is the time of the first reported case or the time of root node in the phylogeny, whichever comes first.

Estimates of the reporting rate and the epidemic start date were not affected by assumptions of k, regardless of the data used during inference.

### Estimation from Multiple Phylogenies

**Fig. 3.**
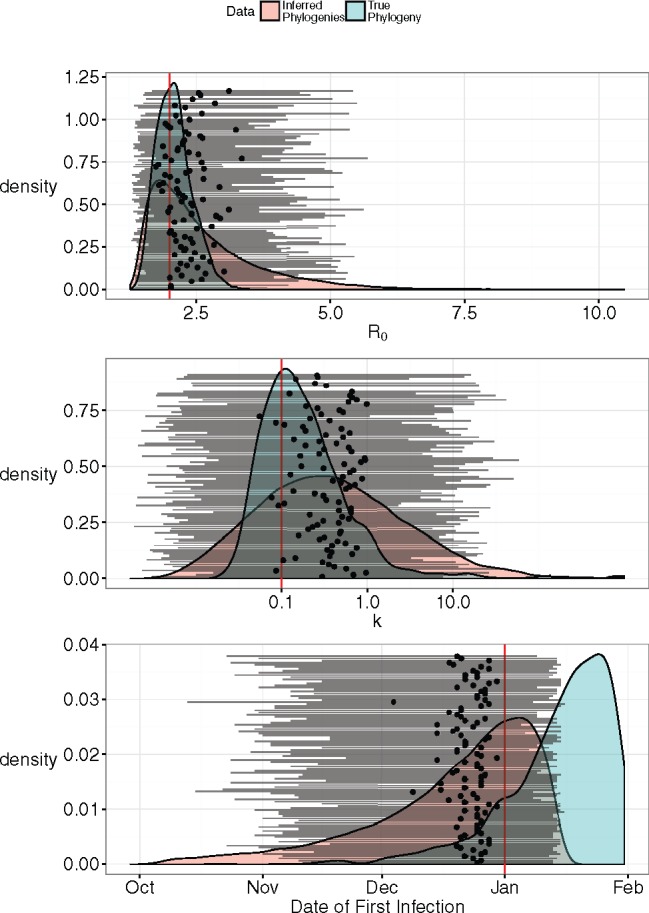
Inference using 100 trees sampled from simulated sequences, compared with the estimates obtained using the true phylogeny. Horizontal lines with a black dot in the middle represent the median and 95% HPD intervals of parameter values estimated using each of the 100 trees. The red vertical lines are the true parameter values. The red distributions are the posterior distributions integrated over the 100 phylogenies, and the blue distributions are the posterior distributions obtained using the true phylogeny.

For a subset of data (those generated using $R_{0}=2$ and k=0.1), we re-estimated the parameters for each phylogeny inferred from the simulated sequences (fig. 3). All 95% HPD intervals obtained using inferred phylogenies included the true parameter value. However, estimates of $R_{0}$ and k obtained from inferred phylogenies instead of the true phylogeny reduced precision and increased bias, although estimates of k were still more precise than those estimated from epidemiological data. Interestingly, estimates of the epidemic start date were less biased when using inferred phylogenies than the true phylogeny, although this might simply be due to the sample phylogeny randomly having a tree height further away from the epidemic start date.

### Phylodynamic Analysis of a Wild Poliovirus Type 1 Outbreak

Although most poliovirus infections are asymptomatic, temporary or permanent paralysis can occasionally occur. Those symptomatic cases are reported to the World Health Organization. Accurately estimating the reporting rate (i.e., case-to-infection ratio) is especially important as the eradication of polio approaches completion. The consensus value often used in epidemiological modeling of wild poliovirus type 1 (WPV1) is 0.5% (Grassly etal. 2006; Blake etal. 2014). If the reporting rate is lower than expected, then a much longer period of no reported infections must pass before eradication can be certain (Eichner and Dietz 1996; Kalkowska etal. 2015). We wanted to apply our inference method to poliovirus phylogenies to see if we can obtain more accurate estimates of the reporting rate compared with using incidence time series alone.

**Fig. 4.**
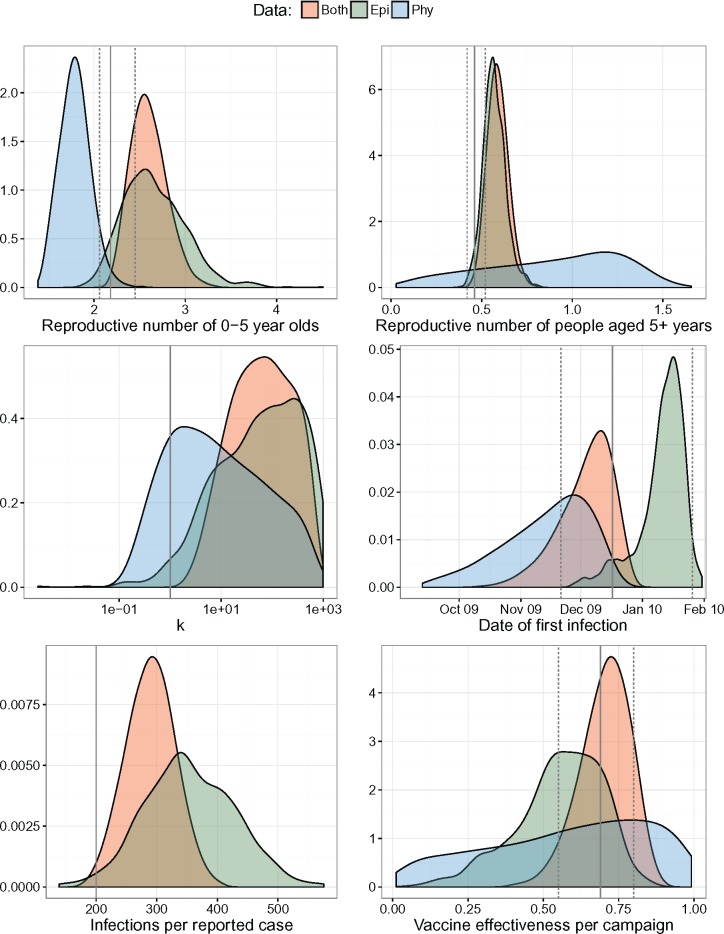
Posterior densities of epidemiological parameters for the 2010 wild type 1 poliovirus outbreak in Tajikistan. The solid and dashed vertical lines are the maximum likelihood estimates and 95% confidence intervals estimated in Blake et al (2014) using epidemiological data only. The solid vertical lines not accompanied by dashed lines correspond to parameter values that were fixed and not estimated.

In 2010, a large outbreak of wild poliovirus type 1 occurred in Tajikistan resulting in 518 reported cases of poliomyelitis (Yakovenko etal. 2014). Fitting an SEIR model structured by three age groups to incidence time series, Blake etal. (2014) used iterated filtering to obtain maximum likelihood estimates of various epidemiological parameters. We fit the same model to pathogen phylogenies, incidence time series, or both using a Bayesian approach. The posterior distributions of parameters are presented in figure 4, and the median and 95% HPD intervals are in supplementary table S1 in the Supplementary Material online.

The rate of substitution estimated using MrBayes was 0.010 (0.006, 0.014) substitutions per site per year, which was in line with previous estimates (Jorba etal. 2008).

We obtained more precise estimates of the reporting rate when using both epidemiological and phylogenetic data. These estimates are dependent on the initial number of susceptible individuals, which we fixed to their maximum likelihood estimates obtained by fitting to epidemiological data only (Blake etal. 2014).

The basic reproductive number of children aged 0–5 years $R_{c}$ was estimated to be 2.58 (2.23–2.98) when both epidemiological and phylogenetic data were used in inference. These values were more similar to the results obtained from just the incidence time series than those estimated from pathogen phylogeny, indicating that the epidemiological data were more informative of the reproductive number than pathogen phylogeny. The maximum likelihood estimate of 2.18 from Blake etal. (2014) were included within the 95% highest posterior density (HPD) interval except when only phylogenetic data were used for inference.

The posterior distributions of the reproductive number of older children and adults $R_{a}$ all included the maximum likelihood estimate of 0.46. Adding the phylogenetic data did not significantly alter parameter estimates. This was not surprising as the credible interval surrounding estimates using just phylogenetic data was much wider.

The estimated value of k was high (>1), indicating the lack of superspreading dynamics. The estimated values were 6.7 (0.1–349.7) when only genetic data were used for inference. These values were much higher when epidemiological data were used: 69.0 ($2.6\times 10^{-3}$–729.8), and when both data sets were used at the same time: 64.0 (1.8–518.1).

Given the large credible intervals around estimates of vaccine effectiveness per campaign using phylogenetic data, only epidemiological data were informative of this parameter. The credible intervals using just epidemiological data included the maximum likelihood estimate from Blake etal. (2014) at 69% (55–80%).

Finally, the estimated start date of the epidemic for analysis using both data sets, epidemiological data only, and phylogenetic data only all overlapped with each other, as well as with estimates from Blake etal. (2014).

Overall, it seems that incidence time series were more informative for estimates of reproductive number, k, and vaccine effectiveness than pathogen phylogeny. However, in all these cases, including the pathogen phylogeny improved the precision of estimates.

## Discussion

Building on methods that enable parameter inference for stochastic models and phylodynamic approaches integrating both epidemiological and phylogenetic data, we presented a framework for quantifying the offspring distribution dispersion k while inferring key epidemiological parameters from both types of data. The addition of pathogen phylogeny to epidemiological inference was necessary to accurately estimate the dispersion of the offspring distribution k. This would be useful for detecting superspreading dynamics in infectious disease outbreaks where data from densely sampled transmission networks are not available.

The phylogenetic data were not useful for all estimated parameters, however. In the poliovirus analysis, phylogenetic data alone were not informative of vaccine effectiveness per campaign or the basic reproductive number of older children and adults.

The use of a single representative phylogeny to infer epidemiological parameters is sufficient for well-resolved phylogenies. Rasmussen etal. (2014) found that parameters of an HIV transmission model were broadly consistent amongst 10 phylogenies sampled from Bayesian phylogeny reconstruction in BEAST. As these sequences were collected over a number of years, there was sufficient confidence in the branching times that different phylogenies sampled from the posterior distribution produced similar estimates. In an outbreak setting when transmission happens over a short time compared with viral evolution, greater uncertainty in branching times meant that we needed to use a larger number of phylogenies to be confident of the posterior distribution of parameters. For the analysis of the 2010 polio outbreak in Tajikistan, integrating over a large number of phylogenies was necessary given the extent of uncertainty in branching times (supplementary fig. S4, Supplementary Material online).

Ideally, an inference framework would concurrently estimate epidemiological parameters and reconstruct the phylogeny. This could be implemented in existing phylogenetic reconstruction packages such as MrBayes (Ronquist etal. 2012) and BEAST (Drummond etal. 2012; Bouckaert etal. 2014) by incorporating a particle filter. However, such a framework would be even more computationally intensive than PMCMC due to the additional parameters that need to be estimated.

Existing approaches to epidemiological inference from pathogen phylogeny do not usually account for overdispersion in the offspring distribution (Volz etal. 2009). While the variance of the offspring distribution can be increased by dividing the population into a limited number of infectious categories (Volz and Pond 2014), the number of secondary infections per individual lies on a continuum in real populations. Also, discretization of infectiousness requires a structured coalescent approach whereas estimating the offspring distribution parameters assumes homogeneous mixing.

In previous studies on the relationship between the effective population size $N_{e}$ and the prevalence N have assumed that $N_{e}=\frac{N}{\sigma^{2}}$ (de Silva etal. 2012; Magiorkinis etal. 2013). However, this relationship is only accurate whenN is constant. The formulation used in this paper is valid for any arbitrary time-varying offspring distribution and changing prevalenceN (Fraser and Li 2017).

Although the particle filter produces an unbiased estimate of marginal likelihood, it is very computationally intensive. The number of particles required scales with the length of simulations, the number of transitions in the model, and the reporting probability of cases. As we simulated from the index case, the epidemic trajectories at the beginning of simulations were highly unpredictable. Data sets with overdispersed offspring distribution further increased the stochasticity of simulations, necessitating a large number of particles to obtain a stable estimate of the marginal likelihood. In our implementation, we needed 10,000 particles for k=0.1 and at least 1,000 for k=1. For simpler models, approximations such as the Kalman filter can be used. The strength of PMCMC, however, is the applicability to a wide range of models including high-dimensional ones (Sheinson etal. 2014).

We provide a parallelized C ++ implementation of the PMCMC algorithm with an accompanying R package to process input and output for the C ++ program. We decided to use our own implementation of PMCMC as existing programs and libraries did not provide all the necessary features we needed for inference from both epidemiological and phylogenetic data. The R package POMP provides an extensive array of inference and simulation methods for Markovian processes including PMCMC (King etal. 2016), however the program is not easily parallelizable and is not well suitable for inference from phylogenetic data. On the other hand, the BEAST packages are well optimized for inference of epidemiological and evolutionary parameters from pathogen sequences but have not yet implemented particle filtering and have a limited number of epidemiological models (Drummond etal. 2012; Bouckaert etal. 2014).

In addition to methodological contributions, we demonstrated the value of a phylodynamic approach for poliovirus research. While phylodynamic analyses have been used to characterize the epidemiological dynamics of other viral diseases such as influenza and HIV, such methods are not widely used for poliovirus analysis. Molecular surveillance through sequencing of poliovirus isolates has mainly been used for tracking the geographic spread of poliovirus in endemic countries (Angez etal. 2012), detecting orphan lineages which are indicative of long-term silent transmission (Gumede etal. 2014), and reconstructing the history of pathogen diversity (Burns etal. 2013). Although model-based parameter inference has been used to analyze epidemiological data for polio (Grassly etal. 2006; Mangal etal. 2013; Blake etal. 2014), it has not been used to analyze viral sequence data.

The gold standard of polio surveillance has traditionally been through Acute Flaccid Paralysis (AFP) surveillance, in which stool samples from patients with AFP symptoms are tested for the presence of poliovirus. As the number of poliovirus infections decreases, there might be too few symptomatic cases reported through AFP surveillance to provide sufficient data in terms of incidence time series and viral sequences. Environmental sampling of poliovirus shed by asymptomatically infected individuals will thus play an increasingly important role in monitoring poliovirus and quantifying its epidemiology as eradication gets closer.

The inference framework presented here integrates the analyses of epidemiological and phylogenetic data. It can account for demographic stochasticity and phylogenetic uncertainty to quantify heterogeneity between individuals at the same time as estimating other epidemiological parameters. This inference method can be further applied to other rapidly evolving viral infections especially those with superspreading dynamics.

## Materials and Methods

### Coalescent Likelihood Calculation

The likelihood given a phylogeny (Phy) is calculated in a piecewise fashion for small time intervals. The small time intervals are unequal in size, and bounded by the times for one of three “events”: end of a simulation time step (total of $n_{T}$ steps), coalescence (total of $n_{tips}-1$events), or sampling (total of $n_{tips}$ events). The length of each time interval between events is denoted by $U_{s}$ where $s=\{1,\text{…},n_{T}\text{+}2n_{tips}-1\}$ and the number of lineages at the end of each interval is $A_{s}$. Let g(t) return a vector of indices of time intervals the phylogeny corresponding to simulation time step t. The phylogenetic data at simulation time step t are summarized by ${\text{Phy}}_{t}=\{U_{g(t)},A_{g(t)}\}$.

At each simulation time step t, we calculated the coalescent likelihood sequentially for each time interval s∈g(t),

(3) $$P({\text{Phy}}_{t,s}|\lambda_{t,s})=\{\begin{array}{c} \left(\begin{array}{c} A_{s}\\ 2 \end{array}\right)\lambda_{t}e^{-\left(\begin{array}{c} A_{s}\\ 2 \end{array}\right)\lambda_{t}U_{s}}ifintervalsstartswithacoalescentevent\\ e^{-\left(\begin{array}{c} A_{s}\\ 2 \end{array}\right)\lambda_{t}U_{s}}otherwise \end{array},$$

where $\lambda_{t}$ is calculated from the simulated epidemic trajectory using equation (1). The overall likelihood for all time intervals in simulation time step t is the product of probabilities calculated using equation (3)$P\left({Phy}_{t}|\lambda_{t}\right)=\prod_{s}P({Phy}_{t,s}|\lambda_{t,s})$ for s∈g(t).

For the poliovirus analysis we used an SEIR model instead, which included a short latent compartment E after an individual became infected. Because we still simulated secondary infections at the end of an individual’s infectious period, this was equivalent to an SIR model with a gamma-distributed instead of exponentially distributed generation time. Because the latent period was very short compared with the infectious period, we approximated the coalescent likelihood with the same formula as above, replacing $I_{t}$ with $E_{t}+I_{t}$ and setting $T_{g}$ as the duration of infection.

We ignored age-structure in the likelihood calculation based on the phylogeny because mixing between age groups occurred at a sufficiently high rate that we could ignore population structure. For the purposes of inference, we calculated the likelihood for the phylogeny using the reproductive number for the whole population. However, we included age-structure in the epidemic model because different age groups were vaccinated at each of the three immunization campaigns.

When data are sparsely sampled, the epidemiological and phylogenetic data can be considered to be independent. Thus, when inferring from both types of data, the overall likelihood is calculated as the product of epidemiological and phylogenetic likelihoods.

### Particle MCMC Procedure

The aim of the statistical inference framework presented here is to obtain the Bayesian posterior distribution P(θ|D)∝P(D|θ)P(θ), where $\theta =(\theta_{1},\ldots ,\theta_{n_{\theta }})$ is a vector of $n_{\theta }$ parameters with parameter space $\Theta =(\Theta_{1}^{},\ldots ,\Theta_{n_{\theta }}^{})$. The prior probability P(θ) is updated with the likelihood of parameters given the data D. For compartmental transmission models, it is not usually possible to analytically solve the likelihood function. To solve this problem, particle filtering has been implemented within an Markov chain Monte Carlo (MCMC) framework to estimate the likelihood by integrating over stochastic epidemic trajectories (Andrieu etal. 2010). A stochastic model simulation generates an epidemic trajectory $X_{0:n_{T}}$ from time step 0 to $n_{T}$ describing the temporal changes in incidence, prevalence, and reproductive number. Initial model conditions are given by $X_{0}$. Data comprise incidence time series and phylogenetic data $D_{1:T}=\{{Epi}_{1:n_{T}},{Phy}_{1:n_{T}}\}$. ${Epi}_{t}$ refers to the total number of reported cases during the time interval [(t-1) Δt,tΔt].

**Fig. 5.**
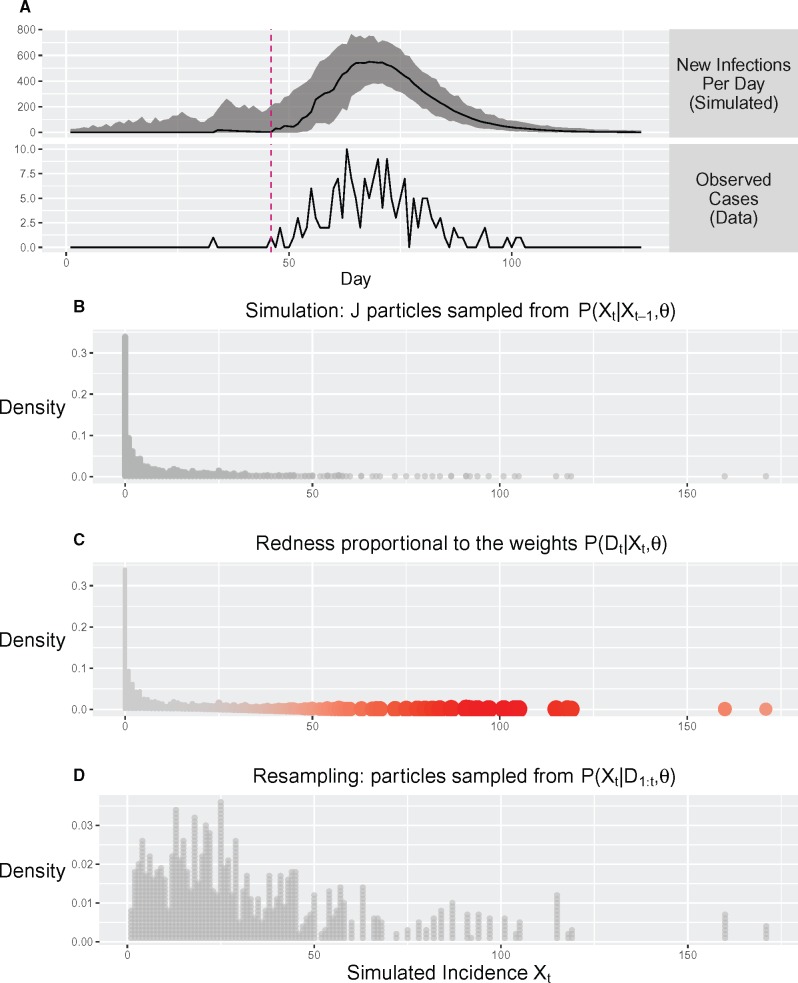
Illustration of likelihood estimation using particle filtering (PF). (*A*) The median and range of simulated epidemic trajectories during PF. (*B–D*) show the steps that occur during one iteration of PF. (*B*) J epidemics (particles) are simulated. The frequency distribution of the simulated $X_{t}$ is proportional to the probability density $P(X_{t}|X_{t-1},\theta )$. (*C*) The weight of each simulated epidemic (particle) is calculated according to the likelihood $P(D_{t}|X_{t},\theta )$. (*D*) Particles are resampled with replacement according to multinomial distribution where probabilities are the normalized particle weights. Further details of the PF implementation are given as pseudocode and discussed in more detail in the “Materials and Methods” section.

The overall marginal likelihood is calculated sequentially for each discrete time step indexed by $t=\{1,.,n_{T}\}$ (eq. 4; fig. 5).

(4) $$\begin{array}{cc} & P(D_{1:n_{T}}|\theta )=\int^{P}(D_{n_{T}}|X_{0:n_{T}},\theta )P(X_{0:n_{T}}|\theta )dX_{0:n_{T}}\\ & {=}^{\int }\prod_{t=1}^{n_{T}}[P(D_{t}|X_{t},\theta )]P(X_{0}|\theta )\prod_{t=1}^{n_{T}}[P(X_{t}|X_{t-1},\theta )]dX_{0:n_{T}} \end{array}$$

Pseudocode of the inference procedure is provided below. We assume that both epidemiological and phylogenetic data are used.

1. Sample M phylogenies indexed by *m* from the posterior distribution of a Bayesian phylogenetic reconstruction program ${Phy}_{1:n_{t}}^{\left(m\right)}$.

FOR each phylogeny m in 1 to M

2. Calculate marginal likelihood $L:=P(D|\theta^{init})$ using particle filtering and set $\theta :=\theta^{init}$. (See particle filtering algorithm below)

FOR iteration i in 1 to MCMC iterations

3. Propose new parameter values $\theta^{*}:=q(\theta^{*}|\theta )$ where q is the proposal distribution.

4. Calculate the marginal likelihood $L^{*}=P(\theta^{*}|D)$ using the particle filtering algorithm below.

5. Calculate acceptance probability of new parameters $p_{a}=\frac{q(\theta |\theta^{*})P(\theta^{*})P(D|\theta^{*})}{q(\theta^{*}|\theta )P(\theta )P(D|\theta )}$

6. Draw a random number z∼Unif(0,1). IF $\left(z<p_{a}\right)$ THEN $\theta :=\theta^{*}$ and L:=L* ELSE $\theta :=\theta^{}$

END LOOP

7. Remove first 50% of samples as burn-in and sample every x iteration from θ values accepted by MCMC.

END LOOP

8. Concatenate samples from all phylogenies. Calculate the median and 95% highest posterior density intervals.

The particle filtering algorithm used to calculate the marginal likelihood is given below. J is the number of particles, where each particle is associated with an epidemic trajectory $X_{0:n_{T}}^{\left(j\right)}$, and a particle weight $\omega_{}^{\left(j\right)}$. The epidemic trajectory comprises two state variables that vary with time: incidence and pairwise coalescent rate.

FOR time step t in 1 to $n_{T}$

FOR particle j in 1 to J

1. Simulate $X_{t}^{\left(j\right)}$ according to model.

2. Set the weight to the likelihood $\omega_{}^{\left(j\right)}:=P(D_{t}|X_{t}^{\left(j\right)})$.

END LOOP

3. Calculate the mean weight ${\bar{\omega }}_{t}:=\frac{1}{J}\sum_{j=1}^{J}\omega_{}^{\left(j\right)}$.

4. Use a multinomial distribution with probabilities $\Omega_{}^{\left(j\right)}=\frac{\omega_{}^{\left(j\right)}}{\sum_{i=1}^{J}\omega_{}^{\left(j\right)}}$ to resample J particles for the next time step.

END LOOP

5. Calculate the marginal likelihood $L(\theta |D_{1:T})=\prod_{t=1}^{n_{T}}{\bar{\omega }}_{t}$.

END LOOP

For incidence time series, the likelihood calculation uses the probability mass function of the binomial: P(Epit|Xtj)=XtjEpitρEpitj(1-ρ)Xt(j)-Epit, whereρ is the probability of a case being reported. If the simulation time step is smaller than the reporting period for incidence data, then we only calculate the likelihood every x number of simulation time steps, such that xΔt is equal to or greater than the reporting period.

At the start of an MCMC chain, the initial parameter values were set to their true parameter values in the case of simulated data to reduce convergence time. We simulated an additional 100 data sets from the stochastic SIR model with true parameter values $R_{0}=2$, k=1, and ρ=1%, but starting the MCMC chains at parameter values far from their true values. Using a heated chain at the beginning of the MCMC (multiplying $p_{a}$ by a factor), we found that the MCMC chain converged on the same posterior distributions as when the initial parameter values were close to the true parameter values (supplementary table S2, Supplementary Material online).

For the poliovirus analysis, the initial parameter values were set to those obtained through by fitting a stochastic SEIR model to incidence time series (Blake etal. 2014).

For the simulated data, up to 500,000 MCMC iterations were carried out, sampling parameter values every 100 iterations. Convergence was determined by calculating the effective sample size after removing the first 50% of samples as burn-in. Samples with an effective sample size <200 were removed from the final result plot.

At each iteration of the MCMC, we used a Gaussian distribution $q(\theta_{i}^{*}|\theta_{i})$ to propose a new parameter value $\theta_{i}^{*}$ centered around the old parameter value $\theta_{i}$, where $i=\{1,\ldots ,n_{\theta }\}$ and $n_{\theta }$ is the total number of parameters to be estimated. For k, we estimated its reciprocal $\frac{1}{k}$ so the proposal distributions were centered around $\frac{1}{k}$ instead. We did not include a covariance matrix because we proposed a new value for only one parameter at each iteration of MCMC.

The standard deviation $\sigma_{i}$ of proposal distribution $q(\theta_{i}^{*}|\theta_{i})$ was adjusted to optimize the acceptance probability of parameter $\theta_{i}$ During the first 20,000 proposals of a parameter $\theta_{i}$, the acceptance probability of the parameter $a_{i}$ was calculated every 200 proposals. If $a_{i}<0.15$ or $a_{i}>0.75$, the standard deviation of the proposal distribution $\sigma_{i}$ was reduced or increased, respectively. Assuming an optimal acceptance probability $a_{opt}=0.234$ (Roberts and Rosenthal 2001), the standard deviation was adjusted using:

(7) $$\begin{array}{c} \sigma_{i}^{\text{*}}=\sigma_{i}e^{\frac{1}{2}(a_{i}-a_{opt})}. \end{array}$$

The time per MCMC iteration depends on the length of the time series data, the number of particles, and the number of random number draws per simulation time step. For a simulated data set with around 130 time steps, it took 0.77 seconds per MCMC iteration on a Linux cluster with 20 cores (Imperial College High Performance Computing Service) using 20,000 particles and both incidence time series and pathogen phylogeny for inference. The number of particles depended on the length of the time series, the number of compartments in the model, and the stochasticity of the model. For example, simulations are more stochastic when *k* is small so more particles are needed to prevent particle depletion. An example is provided in supplementary figure S5 in the Supplementary Material online to illustrate the issue of particle depletion when simulating outbreaks.

### Overview of Simulation Study

We tested the PMCMC inference framework on simulated data first to determine the accuracy of parameter estimates, assess the value of phylogenetic data in epidemiological inference, and to demonstrate the importance of estimating k. For the simulation study, we generated 60 simulated data sets using a stochastic SIR model under various combinations of $R_{0}$, k and reporting probability ρ (see supplementary figs. S2 and S3, table 3, and Supplementary Information in the Supplementary Material online for more details on the simulations). Each data set comprised a phylogeny and an incidence time series for a sample of infected individuals. The phylogeny is a dated phylogeny representing the genealogy of the sampled individuals. For each data set, we performed three sets of inference: using incidence time series; using phylogenetic data; or using both. The following parameters were concurrently estimated: $R_{0}$, $T_{g}$, k, $T_{0}$, and ρ when incidence time series were used during inference. The results were shown in figure 1, supplementary figure S1 in the Supplementary Material online, and table 1.

To assess the consequences of not estimating k, we re-estimated all other parameter values except k for 30 of the simulated data sets (those with ρ=1% sampling) while fixing k=1. Again, we conducted statistical inference 3 times using one or both sets of data. The posterior estimates were shown in figure 2.

For one of the simulated data sets ($R_{0}=2$, k=0.1), we simulated the evolution of pathogen sequences down the true phylogeny to obtain a sample of pathogen sequences. Using MrBayes (Ronquist etal. 2012) to reconstruct the phylogeny from the pathogen sequences, we obtained a posterior distribution of phylogenies given the simulated pathogen sequences. We then sampled 100 phylogenies from this posterior distribution and re-estimated the parameter values using each sampled phylogeny. The posterior estimates using all phylogenies were shown in figure 3.

For all the parameter estimation above, we set the initial parameter values to be very close to the true parameter values (those used to simulate the data). To test that we can obtain the true parameter values in the absence of prior information, we generated 100 extra simulated data sets using $R_{0}=2$, k=1 and ρ=1%. For each of these, the initial parameter values were randomly sampled from the prior distributions for the parameters. The coverage, precision, and bias of estimates are presented in supplementary table S2 in the Supplementary Material online.

### Incorporation of Phylogenetic Uncertainty

Using a fixed phylogeny to infer parameters would not cause problems if confidence in the branching times was high. However, low diversity among pathogen sequences increases the uncertainties in parameter estimates due to uncertainties in topology and branching times.

For one of the outbreaks simulated using $R_{0}=2$ and k=0.1 and sampled with 1% probability, we simulated sequence evolution down the sampled phylogeny using seq-gen (Rambaut and Grassly 1997). In addition to the sampled sequences, we also simulated the sequence evolution of an outgroup so that the tree could be rooted. Each sequence was 1,000 nucleotides in length with equal equilibrium frequencies of A, C, T, and G. We used the JC69+Γ model of substitution (Jukes and Cantor 1969) with a rate of substitution of 0.15 per site per year. This ensured that sufficient evolution would take place during the outbreak.

We used MrBayes (Ronquist etal. 2012) to estimate the phylogeny from the simulated sequences assuming a strict molecular clock and a JC69+Γ model. We used the outgroup sequence to root the phylogenies, and the tip sampling dates to estimate the rate of nucleotide substitution. From the resulting posterior distribution, we sampled 100 dated phylogenies. We divided the branch lengths of phylogenies measured in substitutions per site by the estimated molecular clock rates to obtain dated phylogenies. For each dated phylogeny, we re-estimated the epidemiological parameters. An overall posterior distribution was obtained by concatenating samples of parameter values obtained for each phylogeny.

### Assessing the Coverage, Bias, and Precision of Estimates

The coverage was determined by the percentage of simulations for which the true parameter value was within the 95% HPD interval of estimates. Bias was the distance between the median parameter estimate and the true parameter value. Finally, the precision was determined by the Root Mean Squared Deviation (RMSD) using the formula $\sqrt{\frac{1}{n}\sum_{i=1}^{n}(\theta_{i}-\hat{\theta }{)}^{2})}$, where $\theta_{i}$ for i={1,.,n} were the n values of parameter θ sampled from the posterior distribution of the parameter and $\hat{\theta }$ was the true parameter value.

### Poliovirus Analysis

Data used for poliovirus outbreak analysis were collected during the 2010 outbreak of WPV1 in Tajikistan, which resulted in 518 confirmed and polio-compatible cases (CDC 2010). Poliovirus genomes in stool samples collected from patients were sequenced in the 960-nucleotide VP1 region (Yakovenko etal. 2014). A total of 116 sequences were obtained from the stool samples in Tajikistan (GenBank: KC880365–KC880521). Each sequence was associated with the date of collection.

The posterior distribution of phylogenies was estimated using MrBayes (Ronquist etal. 2012), assuming a K80+Γ model of substitution (Kimura 1980) and a strict molecular clock. A uniform prior was placed on the branch lengths to avoid specifying a population model when inferring the phylogeny, given that the phylogeny would then be used to infer population dynamics. The K80+Γ model was selected by as it returned the lowest Bayesian Information Criterion score in jModelTest2 (Guindon and Gascuel 2003; Darriba etal. 2012). Phylogenies were rooted using an outgroup sequence sampled in India in 2009 (GenBank: KC800662). The tip dates were fixed to the date of sampling that is, the date of first stool collection. This was usually within 48 h of a patient arriving with symptoms of acute flaccid paralysis (AFP). As with the simulated data, dated phylogenies were obtained by dividing the branch lengths of reconstructed phylogenies by the molecular clock rate. We sampled 100 dated phylogenies from the MrBayes posterior and estimated parameter values based on each phylogeny.

A time series of daily reported cases was constructed from the line list and formed the epidemiological data. This data set was divided into three age groups: 0–5, 6–14, and 15+ years, which corresponded to the target age groups of supplementary immunization activities (CDC 2010).

We fit the SEIR model used in Blake etal. (2014) to the polio data. The model is divided into three age groups indexed by i. $S_{t,i},E_{t,i},I_{t,i}$ and $R_{t,i}$ are the number of susceptible, exposed, infectious and recovered individuals in age group i at time t. Details of the model can be found in the Supplementary Material online. Unlike the usual SEIR model, all infections occurred at the end of an individual’s infectious period. Thus the model used in this paper can be considered a SIR model with gamma-distributed generation time.

We fixed the initial susceptible population sizes to those used in Blake etal. (2014), and also placed a strong prior on the duration of infectiousness based on likelihood profile obtained in Blake etal. (2014). Fixed parameter values and prior distributions on estimated parameters are outlined in table 4.

**Table 4.** Model Parameters of the Transmission Model for Polio.

Parameter	Value	Estimated	Prior
Population sizes in thousands $N_{\text{Total},1},N_{\text{Total},2},N_{\text{Total},3}$	656, 1,249, 3,721		
Susceptible individuals at start in thousands $S_{0,1},S_{0,2},S_{0,3}$	109.6, 176.1, 104.2		
Initial numbers of infected $I_{0,1},I_{0,2},I_{0,3}$	1, 0, 0		
Mean duration of latency $T_{L}=\frac{1}{\gamma_{1}}$	4		
Mean duration of infectiousness $T_{I}=\frac{1}{\gamma_{2}}$		Yes	Gamma (α=5.12,β=1.7)
Reproduction numbers of children aged 0–5 years $R_{c}$		Yes* (proposal and prior on β)	
Reproduction numbers of people aged 6+years $R_{a}$ relative to $R_{c}$		Yes* (proposal and prior on $\beta_{p}$)	Uniform (1$\times 10^{-5}$, 1)
Offspring distribution dispersion parameter k		Yes	Uniform (1$\times 10^{-5}$, 1,000)
Infections:Case ratio (inverse of reporting fraction) $\frac{1}{\rho }$		Yes	Uniform (1, 1$\times 10^{6}$)
Time of first infection $T_{0}$		Yes	Uniform (08 Sep 09, 01 Feb 10)
Vaccine efficacy υ		Yes	Uniform (0.0, 1.0)
Mean and shape parameters of the Erlang distributed incubation period ξ, α	16.5 days, 16		

Note.—Values of fixed parameters are given in the column “Value.” For parameters that are estimated, the prior distribution on the parameter is given in the “Prior” column. The population was divided into three age groups: 0–5, 6–14 and 15+ years. The initial numbers of susceptibles were fixed to the maximum likelihood estimates used in Blake et al (2014). Vaccinations took place on the following dates: 06 May, 20 May, 03 Jun, 17 Jun and 17 Jun 2010. On these dates, individuals were moved from the susceptible to the recovered compartment with probability υ. Gamma distributions are parameterized by the shape and scale parameters. *The reproductive numbers $R_{c}$ and $R_{a}$ were calculated from the estimated transmission rate amongst young children β, the relative transmission rate between all other groups $\beta_{p}$, the duration of infectiousness, and numbers of susceptibles.

We used 10,000 particles and up to 150,000 MCMC iterations sampling every 20 iterations. The Markov chains were terminated earlier than 150,000 iterations if estimates of the marginal posterior density had an ESS of at least 100.

## Supplementary Material

Supplementary data are available at *Molecular Biology and Evolution* online.

## Supplementary Material

Supplementary Data
