# Supplementary material for: Quantifying Transmission Heterogeneity Using Both Pathogen Phylogenies and Incidence Time Series
Source: Mol Biol Evol. 2017 Jul 11;34(11):2982–95. doi: 10.1093/molbev/msx195 (PMC5850343; doi:10.1093/molbev/msx195)
Supplement: Supplementary Data [file msx195_supp.zip › msx195_Supp3.pdf]

Supplementary Information

Quantifying transmission heterogeneity using both pathogen phylogenies and incidence time series

Lucy M Li[1,2], Nicholas C Grassly[1]*, Christophe Fraser[1,3,]*

[1]Department of Infectious Disease Epidemiology, School of Public Health, Imperial College London, St Mary's Campus, London W2 1PG, UK

[2]Center for Communicable Disease Dynamics, Harvard T.H. Chan School of Public Health, Boston, MA 02115, USA

[3]Oxford Big Data Institute, Nuffield Department of Medicine, University of Oxford, Oxford OX3 7LF, UK

*Equal contributions

Corresponding author: Lucy M Li, luli@hsph.harvard.edu

Other parameter estimates from simulated data

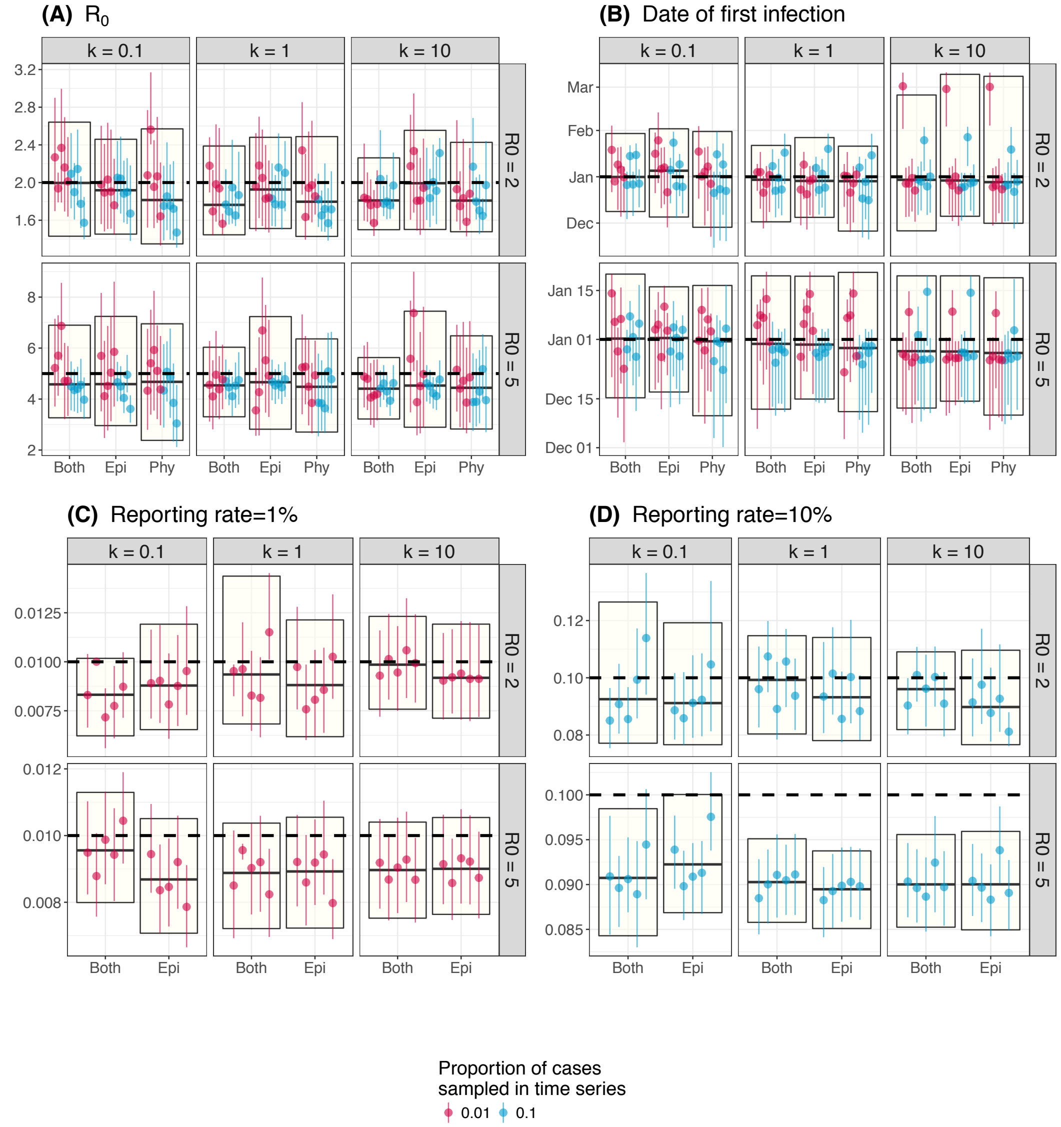

Figure S1. Parameter estimates from simulated data. Estimated values of (A) R0, (B) Date of first infection, and reporting rates for simulations with (C) 1% and (D) 10% sampling rates. The horizontal lines denote the true parameter value for that set of parameters, i.e. the value used to generate the simulated data. The boxes with a horizontal line in the middle indicate the median and 95% HPD interval of parameter estimates pooled from all simulations for that parameter set. The vertical lines with a single dot denote the median and 95% HPD interval of each individual simulation. Blue lines are from simulations in which 10% of individuals were sampled. Red lines are from simulations in which 1% of individuals were sampled. We did not estimate the reporting rate when inferring just from phylogenetic data, as the reporting rate referred to the probability that an infection appeared in the incidence time series. A uniform prior distribution was used for all parameters, with bounds described in Table 3.

Details of simulated outbreaks

We simulated data sets according to an SIR model (see New Approaches section) under 6 combinations of basic reproductive number $R_0 = \{2,5\}$ and $k = \{0.1,1,10\}$. R_0 values of 2 and 5 are reasonable for directly transmitted viral infections (SARS: Fraser et al. 2004; inter-pandemic influenza: Ferguson et al. 2005). Although they do not capture the full range of possible R_0 values for directly transmitted infectious diseases, we were more interested in the impact that different values of k has on parameter estimation. The values of k were selected because they captured the plausible range of values for k. The smallest value of k estimated for infectious diseases is 0.16 for SARS (Lloyd-Smith et al. 2005). Because the implicit assumption of classic SIR models is that the offspring distribution follows the geometric, the scenario of $k = 1$ is considered. The variance of the offspring distribution does not change significantly for $k > 10$, and thus we did not consider values of $k > 10$.

Assuming a constant population size $N = 20{,}000$, the S_t and I_t track the number of susceptible and infectious individuals at time t, while the number of recovered individuals is given by $N - S_t - I_t$. The duration of infection is exponentially distributed with rate $\gamma = 0.2\ \mathrm{day}^{-1}$. The reproductive number at time t was calculated as $R_t = R_0 \frac{S_t}{N}$. More details about the simulated datasets are found in Figures S1 and S2.

Epidemics were simulated with time steps of 0.1 days, starting with 1 infected individual $I_0 = 1$ and $S_0 = 19{,}999$. At each time point t, the number of people recovering was drawn from a binomial distribution $Q_t^{I\to R} \sim Bin(I_t, \gamma dt)$. Another random number was drawn from the negative binomial to determine the number of new infections $Q_t^{S\to I} \sim NBin(R_t Q_t^{I\to R}, k Q_t^{I\to R})$, which translated to a variance of $Q_t^{I\to R} R_t (1 + \frac{R_t}{k})$. This meant that all secondary infections caused by an individual occurred at the end of the infectious period. This approximation was not an issue for these simulations due to the short infectious period; it would not be suitable for analysis of chronic infections.

All parameter values used to generate the parameters, as well as prior distributions used during inference, are listed in Table 3.

Simulations continued until the epidemic died out. We only kept the simulations with a final epidemic size of at least 10.

To generate the observation data we randomly sampled individuals with probability $\rho = \{0.01, 0.1\}$ at the time of recovery. As we tracked who-infected-whom, we reconstructed the dated phylogeny for the sampled individuals. The branching points corresponded to transmission times, and tip dates corresponded to sampling times, i.e. the times of recovery.

R0	k	Days to Epidemic Peak	Total Infected	Sample Size
2	0.1	65	16,284	163
		78	15,817	155
		57	15,534	136
		51	15,423	149
		51	16,354	151
		83	15,759	1586
		99	15,747	1544
		56	16,009	1559
		106	15,915	1577
		64	16,015	1587

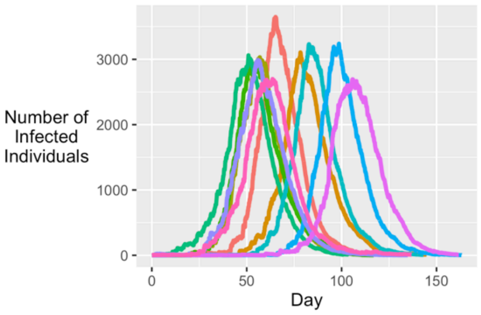

R0	k	Days to Epidemic Peak	Total Infected	Sample Size
2	1	46	15,805	166
		76	15,998	145
		42	15,903	152
		53	16,243	139
		70	15,778	171
		66	15,917	1542
		48	16,176	1631
		55	15,808	1577
		53	16,045	1589
		72	15,745	1611

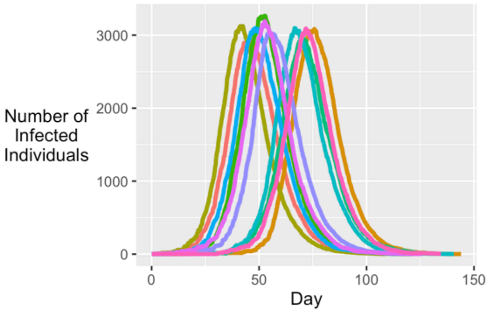

R0	k	Days to Epidemic Peak	Total Infected	Sample Size
2	10	115	15,970	164
		53	15,983	175
		52	15,907	155
		62	15,940	145
		46	16,072	159
		43	15,874	1593
		54	15,969	1633
		92	15,916	1576
		54	16,020	1548
		52	15,873	1551

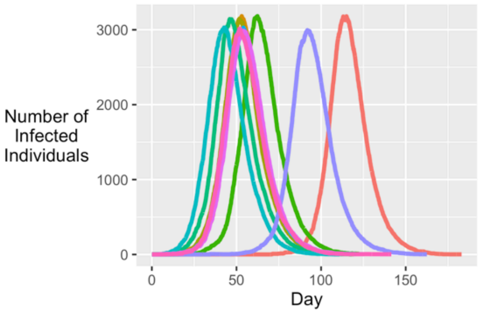

Figure S2. Details and visualization of simulated data when $R_0 = 2$ used in Figure 1.

R0	k	Days to Epidemic Peak	Total Infected	Sample Size
5	0.1	26	19,906	210
		25	19,862	186
		14	19,867	187
		28	19,862	199
		18	19,862	172
		16	19,892	2051
		22	19,863	1971
		24	19,830	1987
		18	19,877	2036
		34	19,849	2084

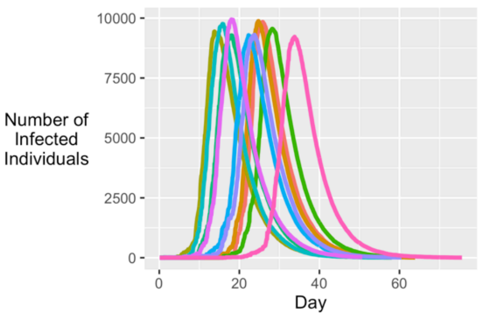

R0	k	Days to Epidemic Peak	Total Infected	Sample Size
5	1	19	19,867	204
		24	19,856	191
		28	19,862	211
		34	19,853	204
		30	19,845	179
		18	19,885	1958
		16	19,858	1964
		16	19,861	1996
		14	19,860	1972
		17	19,851	1952

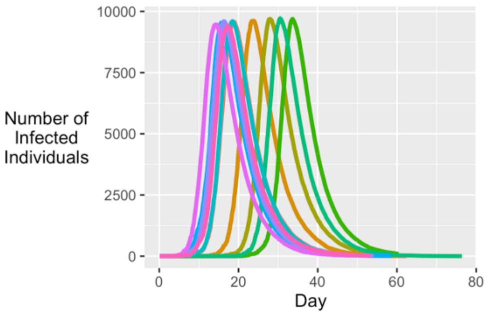

R0	k	Days to Epidemic Peak	Total Infected	Sample Size
5	10	15	19,856	201
		16	19,857	190
		30	19,848	204
		16	19,860	205
		14	19,873	194
		20	19,860	1981
		16	19,856	1976
		14	19,868	1961
		34	19,867	2033
		16	19,854	1966

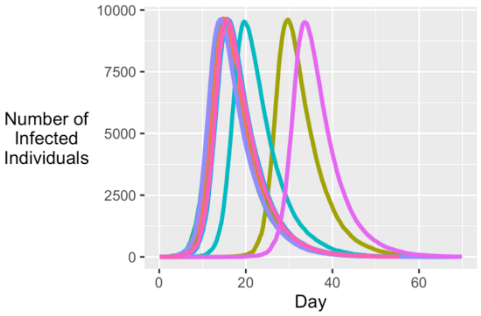

Figure S3. Details and visualization of simulated data when $R_0 = 5$ used in Figure 1.

Tajikistan analysis: model

We fit an SEIR model similar to that used in Blake et al (2014), but with an explicit offspring distribution. Let the transitions between states indexed by age group i be $Q_{t,i} = \{Q_{t,i}^{S_i \to E_i}, Q_{t,i}^{E_i \to I_i}, Q_{t,i}^{I_i \to R_i}, Q_{t,i}^{S_i \to R_i}\}$, where each transition corresponds respectively to a new infection, an infected person becoming infectious, recovery, and vaccination. The transitions are drawn from the following probability distributions

$$Q_{t,i}^{E_i \to I_i} \sim Bin(E_{t,i}, \gamma_1 dt)$$
$$Q_{t,i}^{I_i \to R_i} \sim Bin(I_{t,i}, \gamma_2 dt)$$
$$Q_{t,i}^{S_i \to R_i} \sim Bin(S_{t,i}, V_t v)$$

where γ_1 is the rate of becoming infectious, γ_2 is the rate of recovery, and V_t is an indicator function equal to 1 on the following dates and 0 otherwise: 6 May, 20 May, 3 June, 17 June 2010.

To determine the number of infections, we assumed that all secondary infections caused by each individual occurred at the same time as the generation time was short. The reproductive number of each age group at time t was $R_{t,i} = \frac{1}{\gamma_2} \sum_j \beta_{t,ij} S_{t,j}$. All density-dependent transmission rates $\beta_{t,ij}$ where $i \neq 1$ and $j \neq 1$ were assumed to be a proportion of the transmission rate amongst children of the youngest age group: $\beta_{1,1}\beta_p$. The number of transitions from susceptibles to exposed individuals was drawn from a negative binomial distribution parameterized by its mean and dispersion parameters

$$Q_{t,i}^{S_i \to E_i} \sim NBin(R_{t,i} Q_{t,i}^{I_i \to R_i}, k Q_{t,i}^{I_i \to R_i})$$

Whereas in the simulations we assumed the time of case reporting coincided with the time of recovery, here we could not make that assumption as symptoms may manifest even if an individual is no longer infectious. Instead, we adopted the approach used in Blake et al. (Blake et al. 2014) and modeled the incubation period as an Erlang distribution with mean $\xi = 16.5$ days and a shape parameter of $\alpha = 16$, which was equivalent to the sum of α independent exponential variables with rate $\frac{1}{16.5}$. This distribution was the maximum likelihood fit to data on the length of time between exposure and onset of paralysis (Casey 1942).

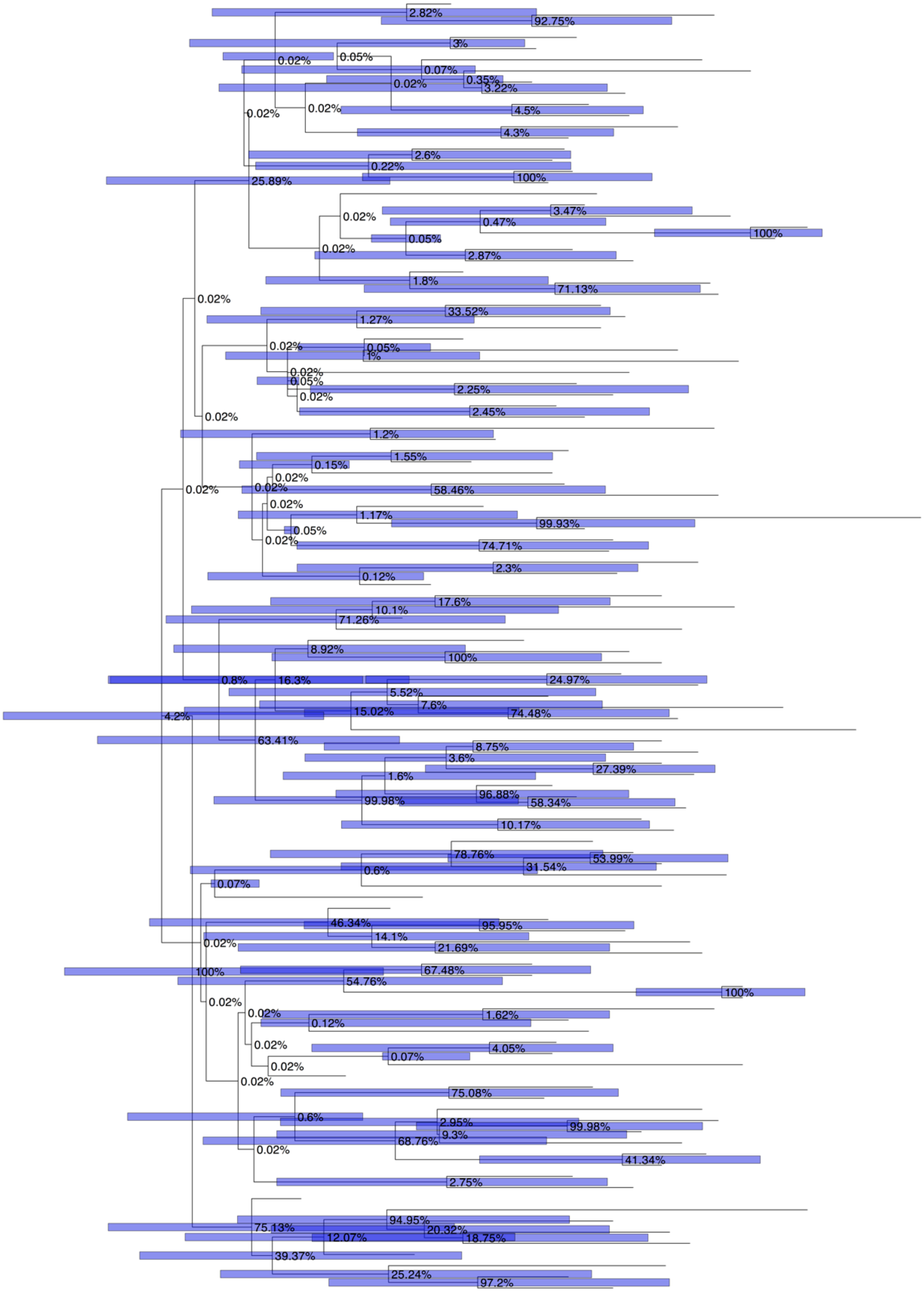

Figure S4. The maximum clade credibility (MCC) phylogeny of 116 wild type 1 poliovirus VP1 sequences from the 2010 Tajikistan outbreak. *The MCC phylogeny was created from the posterior distribution of Bayesian phylogenetic analysis carried out in MrBayes 3.2 (Ronquist et al. 2012). The horizontal bars are the 95% highest posterior density intervals of branching time estimates.*

Table S1. Posterior parameter estimates for the 2010 Tajikistan poliovirus outbreak compared with the maximum likelihood estimates obtained in Blake et al (2014).

**Parameters**	**Both**	**Epi**	**Phy**	**Blake et al. 2014**
R_c	2.58 (2.23-2.98)	2.62 (2.07-3.32)	1.79 (1.46-2.11)	2.18 (2.06-2.45)
R_a	0.59 (0.48-0.7)	0.57 (0.44-0.69)	0.95 (0.15-1.47)	0.46 (0.42-0.52)
k	64.044 (1.842-518.102)	68.956 (0.003-728.782)	6.661 (0.119-349.728)	1
T_0	06 Dec 09 (06 Nov 09-25 Dec 09)	13 Jan 10 (13 Dec 09-27 Jan 10)	13 Jan 10 (13 Dec 09-27 Jan 10)	17 Dec 09 (21 Nov 09-6 Jan 10)
Infections per case	1 in 290 (212-369)	1 in 349 (213-496)	NA	1 in 200
Vaccine effectiveness	71.3% (53.9%-85.9%)	57.1% (24.3%-77.2%)	57.1% (24.3%-77.2%)	69% (55%-80%)

Changing initial parameter values

Given that the true parameter values are not known for real data sets, and that prior information on parameter values are not always available, we added a heated chain before the start of the main MCMC algorithm to find the region of parameter space with high posterior density. When an MCMC chain is heated, the probability that a new parameter value θ^* is accepted p_a is multiplied by a heating factor H_f. This reduces the risk that the MCMC chain remains in a local maximum. We tested this heated chain method on a separate set of simulations to the ones presented in the main text. We simulated 100 data sets from the stochastic SIR transmission model with true parameter values $R_0 = 2$, $k = 1$, and $\rho = 1\%$, but starting the MCMC chains at parameter values far from their true values. During the heated phase, the acceptance probability of parameters was multiplied by 3. After 2000 iterations, the heated chain was cooled by reducing H_f by 0.1 per iteration.

Table S2. The precision (RMSD), bias and coverage (% in HPD) of parameter estimates when the initial parameter values were very far from the true parameter values, averaged across 100 simulations for the parameter combination $R_0 = 2$, $k = 0.1$, and $\rho = 1\%$. *The MCMC chain was initially heated to accept jumps to parameter sets with low posterior densities in order to escape local optima.*

Data	R0: RMSD	R0: Bias	R0: in HPD	k: RMSD	k: Bias	k: in HPD
Both	0.1514	0.0351	100%	0.8970	-0.3504	100%
Epi	0.2009	0.0339	100%	0.9206	-0.7422	100%
Phy	0.1787	0.0672	100%	0.8810	-0.5097	100%
**Data**	**T0: RMSD**	**T0: Bias**	**T0: in HPD**	**ρ: RMSD**	**ρ: Bias**	**ρ: in HPD**
Both	13.1346	2.5898	100%	0.1551	-0.1024	100%
Epi	12.5982	0.1039	100%	0.1795	-0.1174	100%
Phy	14.1991	-0.0382	100%	NA	NA	NA

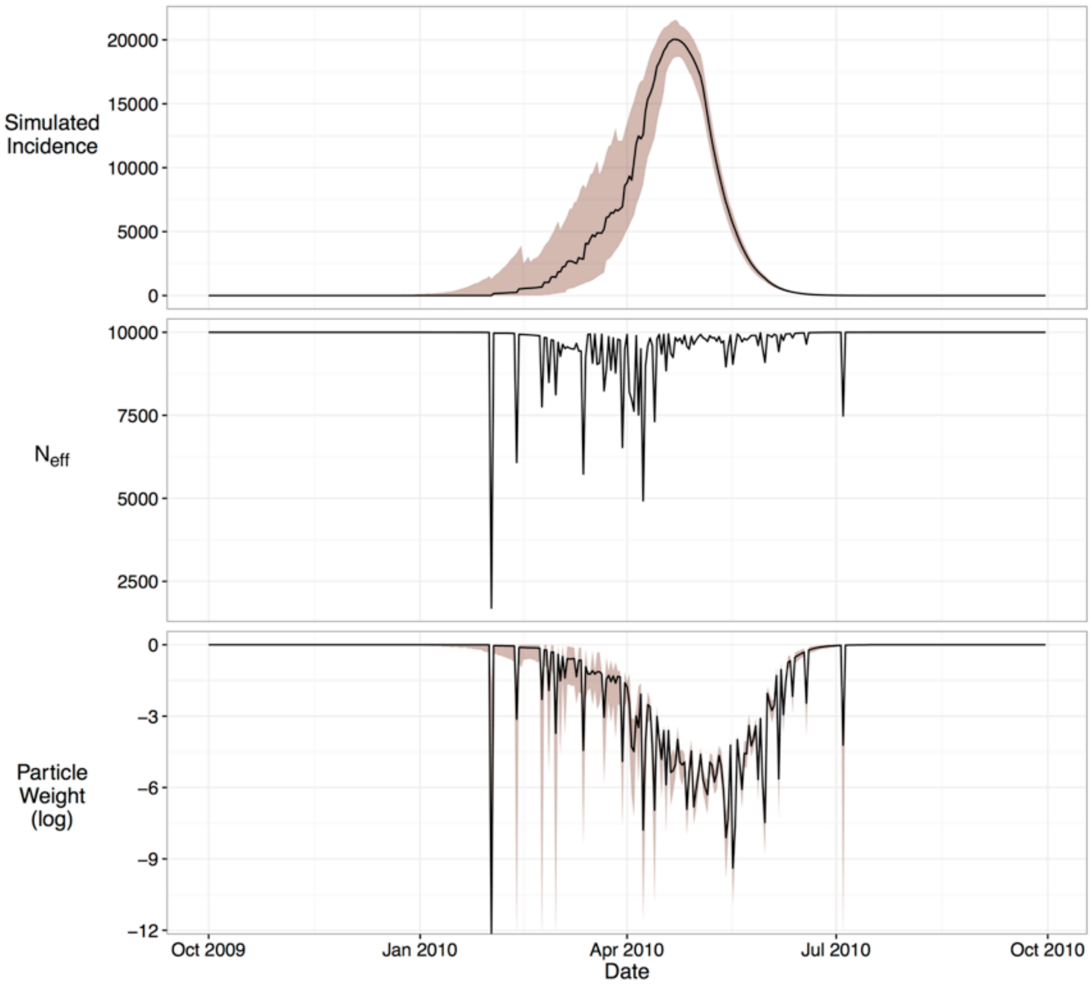

Figure S5. An example of particle filtering during a single MCMC iteration. The median and range of simulated incidence at each time step (1 day) is shown in the top panel. The effective number of particles (N_{eff}) is shown in the middle panel, calculated as $\boldsymbol{N_{eff}} = \frac{\mathbf{1}}{\sum_{j=1}^{J}\left(\boldsymbol{w}^{(j)}\right)^2}$ where each of J particles indexed by j has a particle weight of $\boldsymbol{w}^{(j)}$. The natural log-transformed particle weights at each simulation time step are shown in the bottom panel. The first data point appeared on February 1th 2010, at which point N_{eff} dropped by 80% as most simulations had died out by that point.

References

Blake IM, Martin R, Goel A, Khetsuriani N, Everts J, Wolff C, Wassilak S, Aylward RB, Grassly NC. 2014. The role of older children and adults in wild poliovirus transmission. Proceedings of the National Academy of Sciences 111:10604–10609.

Casey AE. 1942. The incubation period in epidemic poliomyelitis. Journal of the American Medical Association 120:805–807.

Ferguson NM, Cummings DA, Cauchemez S, Fraser C, Riley S, Meeyai A, Iamsirithaworn S, Burke DS. 2005. Strategies for containing an emerging influenza pandemic in Southeast Asia. Nature 437:209–214.

Fraser C, Riley S, Anderson RM, Ferguson NM. 2004. Factors that make an infectious disease outbreak controllable. Proceedings of the National Academy of Sciences of the United States of America 101:6146–6151.

Lloyd-Smith JO, Schreiber SJ, Kopp PE, Getz WM. 2005. Superspreading and the effect of individual variation on disease emergence. Nature 438:355–359.

Ronquist F, Teslenko M, Mark P van der, Ayres DL, Darling A, Höhna S, Larget B, Liu L, Suchard MA, Huelsenbeck JP. 2012. MrBayes 3.2: Efficient Bayesian phylogenetic inference and model choice across a large model space. Systematic Biology 61:539–542.

Volz EM. 2012. Complex population dynamics and the coalescent under neutrality. Genetics 190:187–201.
